# COVID-19 related fears and adherence to infection control protocol amongst immunocompromised transplant recipients: A case study

**DOI:** 10.12669/pjms.38.8.5590

**Published:** 2022

**Authors:** Amena Moazzam, Hafeeza Naz, Khadija Irfan, Zulaikha Mahmood

**Affiliations:** 1 Dr. Amena Moazzam, MRCP (London), FCPS (Medicine), FCPS (Endocrinology), FRCP (London), Department of Endocrinology and Metabolism, Services Institute of Medical Sciences, Lahore, Pakistan; 2 Dr. Hafeeza Naz, FCPS (Medicine), FCPS (Endocrinology), Department of Endocrinology and Metabolism, Services Institute of Medical Sciences, Lahore, Pakistan; 3 Dr. Khadija Irfan, FCPS (Medicine), Department of Endocrinology and Metabolism, Services Institute of Medical Sciences, Lahore, Pakistan; 4 Zulaikha Mahmood, Institute of Administrative Sciences University of Punjab, Lahore, Pakistan

**Keywords:** Adherence, COVID-19, Precautions, Pandemic, Transplant

## Abstract

**Background & Objective:**

Transplant recipients are at a high risk of critical COVID-19 illness due to chronic immunosuppression and their underlying medical condition. Our objective was to study the COVID-19 related fears and adherence to infection control measures in solid organ transplant recipients during COVID-19 pandemic.

**Methods:**

A descriptive study was conducted during the first wave of COVID-19 pandemic (April- August 2020) in Punjab, Pakistan, as a part of province wide COVID-19 awareness drive 754 recipients registered at Punjab Human Organ Transplantation Authority (P-HOTA) for solid organ transplant were contacted telephonically and administered a self-constructed questionnaire. The participants’ response was linked to demographic, anthropometric and disease characteristics available in organizational data base.

**Results:**

Seven hundred fifty four patients who had undergone transplant or were on list during the time period 2018-2020 were identified from data base of PHOTA. 648 patients were contacted while 80 were found to have expired post- transplant and 26 recipients were still on the waiting list. The median age was 31-40 years, with male predominance (5:1). A majority of patients (93%) were found to be well informed about corona infection and its impact on their illness (89%) but (59%) of the recipients had a fear score 25-34(severe) of acquiring infection with female predominance (61%). All the recipients adhered to measures like wearing masks, frequent hand washing and maintaining safe distance .65.9% patient’s preferred online consultation while (77.9%) patients left their shoes outside their house.

**Conclusion:**

During the first wave of COVID-19 pandemic Solid Organ Transplant patients seemed well informed of COVID-19 infection and adhered to precautionary measures against the infections. About 78% of recipients reported that their health and quality of health care (61.1%) during this period remained the same while 3.4% reported of being infected or a contact history with COVID-19 patients

## INTRODUCTION

At present, the global public health is faced with unprecedented threat of dealing with severe acute respiratory syndrome corona virus 2 (SARS-CoV-2) pandemic.^1^,^2^ It has a wide spectrum of symptoms ranging from mild symptoms (81%) to life threatening sepsis and end organ failure (20%). Majority of COVID-19 patients have been observed to suffer from other co morbidities.^3^-^5^ and the elderly male^6^, those who are immune comprised like post-transplant recipients are included in the high-risk group.^7^,^8^

The COVID-19 pandemic has limited the transplantation program in many countries^9^,^10^, and guidelines specific to COVID-19 management in them are under revision as data is limited. Mazzafero et al.^11^ reported a novel experience of long term follow up of three out of 111 liver transplant patients who suffered and died of Corona infection. They were male over the age of 60 years, were diabetic and hypertensive. In the absence of curative treatment, the health organizations have shifted their focus to a model for the containment and prevention of this disease. In addition to isolation of infected cases and their close contacts, it promotes precautionary behaviors among general population.^12^ Previous studies have explored this aspect in different cultural setting with various viral infections.^13^ Reuken et al.^14^ explained the fears and attitudes related to COVID-19 infection in solid organ transplant high risk patients on immunosuppressive treatments during the pandemic 2020 in two German transplant centers.

Our study is an attempt to explore the fears and adherence to infection control measures by post-transplant patients during to the COVID-19 lock down in Pakistan. The COVID-19 pandemic presented as a challenge to the already crippled health care system and struggling economy.^15^ Patients undergoing solid organ transplantation are catered both at public and private sector. Punjab Human Organ Transplantation Authority (P-HOTA) is the official licensing and governing body of transplantation in province of Punjab of Pakistan. The first kidney transplant took place in 1979 in Rawalpindi city^16^, ever since there is a gradual increase in the number of specialized centers of transplantation in the province of Punjab.

### Objective:

The objective was to study the COVID-19 related fears and adherence to infection control measure by immunocompromised post-transplant recipients during COVID-19 pandemic.

## METHODS

A Case study was conducted with data collected from P-HOTA and convenience sampling was used. A total of 754 patients out of 1799 registered cases were approached due to time constraints and inadequacy of data. Eighty patients out of them had expired while 26 were on waiting list for transplant. So, data detail of 648 patients living post-transplant was taken after informed consent. Data collectors were trained in obtaining informed consent and the use of survey instrument. The data was conducted over a period of three months 5th June 2020 to 15th August 2020 in Lahore Pakistan after IRB approval taken from Services Institute of Medical Sciences, No: IRB/2020/678/SIMS

**Fig.1:**
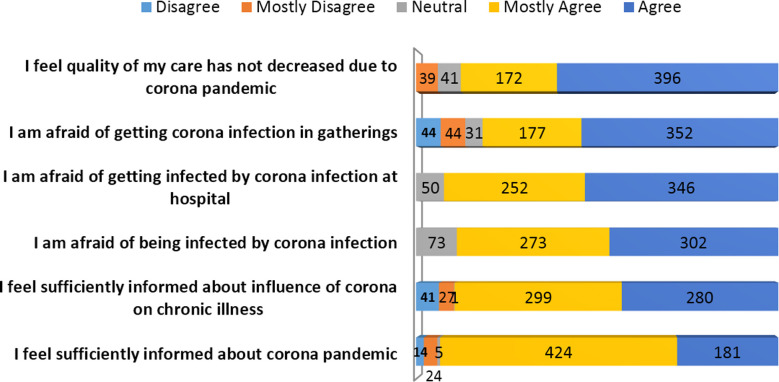
Types of perceived fear.

### Inclusion Criteria:

All those patients aged 11 years and above registered with PHOTA for solid organ transplantation were included. Liver and kidney transplant patients were recruited.

### Primary Outcome:

To identify the COVID-19 related fears and their association with adherence of precautionary measures adopted by the post-transplant recipients during this period.

### Secondary Outcome:

Outcomes of these fear and practices on health of these patients during the COVID 19 pandemic. This was assessed subjectively and by using certain biochemical test. They were asked to compare their health with that prior to COVID-19 pandemic.

### Questionnaire:

The questionnaire of fear and perceptions by Reuken et al^14^ comprised of 6-items was adapted according to local context with reliability score of α = 0.7, which showed acceptable reliability of the instrument. It comprised of 29 questions

Personal characteristics of sample were collected in the following dimensions: gender, age, type of transplant, history of intake of medication and history of co morbid conditions. We also enquired about their symptoms regarding Corona infection and whether they had ever been confirmed of COVID-19 infection or any close relative.

Questions used for assessing fear and perceptions (questions 3, 4, 6, 7 and 10), practices and precautionary measures taken during COVID (6-items questions 11, 12, 13, 14, 15 and 16) and various questions related to health outcomes (questions 1,2,8,9 ,17 and 18) were used.

### Statistical Analysis:

Descriptive statistics were used including frequency and percentages for personal information, fear and perceptions, practices and health outcomes of both pre-transplant & post-transplant. For the relationship between fear and perception and age, Kruskal Wallis test (non-parametric test) was used. For statistical differences between two independent groups (gender & type of transplant) on the basis of fear and perceptions, Mann Whiteny U test was used. To see the relationship between fear and perception with different practices adopted (having two categories Yes and No), Mann Whiteny U test was used.

### Ethical Approval:

(Ref No. IRB/2020/678/SIMS, Dated: 21-07-2020).

## RESULTS

Patients were approached after proper approval from the Director General PHOTA and ethical review board of Services institute of medical health telephonically. The sample had a median age group of 31-40 years with male pre dominance and kidney being the most common type of transplant. Patients profile regarding demographic, anthropometric and associated co morbid conditions is explained in detail (Table-I). The calls were received by the patients themselves (n=518/648,80%) and after proper consent they were asked questions pertaining to their perception, fear and practices. They were asked to compare their present health with that before COVID-19 pandemic while answering the questions. About 93% of patients agreed that their COVID-19 infection related information and its impact on their illness 89% was sufficient. To assess the accuracy of information, certain questions pertaining to cause, mode of spread and preventive measures related COVID-19 infection were asked. They were aware of them being at high risk of severe infection, 3.4% patients gave history of being infected by corona infection and 5.1% recipients gave positive history of contact with covid-19 patient.

**Table-I:** Survey Participants.

A) Demographic Parameters	Frequency	Percentage
***Gender***		
Male	548	84.6
Female	100	15.4
***Age***		
11-20 years	56	8.6
21-30 years	175	27.0
31-40 years	190	29.3
41-50 years	145	22.4
51 and above	82	12.7
Smoker	8	1.2
***Type of hospital***		
Public	187	28.9
Private	461	71.1
***B) Anthroprometric Parametrs***		
BMI		
Underweight (Less than 18.5)	42	6.5
Normal weight (18.5-22.9)	351	54.2
Overweight (23-26.9)	188	29.0
Obese (27 and above)	67	10.3
***C) Diagnosis & Type of Transplant***		
Organs		
Liver	73	11.3
Kidney	575	88.7
***Diagnosis Category***		
ESRD	570	88.0
CLD	66	10.2
Others	12	1.9
Polycystic KD	4	0.6
Malignancy(renal)	8	1.2
***D) Associated Co Morbid Conditions***		
Diabetes	160	24.7
Hypertension	287	44.3
Hepatitis B	56	8.6
Hepatitis C	117	18.1

**Table-II:** Level of Perceived Fear.

Level of Perceived Fear	Moderate fear (Scores: 15-24)	Severe Fear (Scores: 25-34)
Number(n)	266/648 (41%)	382/648 (59%)
***Gender***		
Male	227(41%)	321(59%)
Female	39(39%)	61(61%)
***Age***		
11-20 years	26 (4.01%)	30 (4.63%)
21-30 years	78 (12.03%)	97 (14.97%)
31-40 years	63 (9.72%)	127 (19.6%)
41-50 years	56 (8.64%)	89 (13.73%)
51 and above	43 (6.64%)	39 (6.02%)
***Type of Transplant***		
Liver	34 (5.25%)	39 (6.02%)
Kidney	232 (35.80%)	343 (52.93%)

### Primary outcomes:

### Perceived fear of participants regarding COVID-19:

The perceived fear related to COVID-19 amongst these patients was assessed using the questionnaire with Likert scale. To check the level of perceived fear it was further classified into moderate and severe. Fifty nine percent of patients had severe form of fear related to COVID-19 infection with relatively high percentage in females versus male (61%;59%). The recipients feared most catching infection in gathering especially in hospitals and thus preferred on line consultation with their physicians (65.9%). When these patients we enquired about source of COVID -19 related information, they agreed (n=361)/mostly agreed (n=195) that the source of their information was social media rather than doctors themselves. Further analysis was run to determine an association of these fear and perception to factors like age, gender and type of transplant. It was seen that a significant relation existed between different age groups and emotions like fear and perception X^2^=11.708, p=0.020 < 0.05. However, no significant relationship existed based on type of organ transplant and their gender. On the other hand, there exists a significant relationship between types of hospital (public/private) and fear. Respondents belonged to public hospitals experienced more fear (Mdn=4.5) as compared to private hospitals (Mdn=4.16). It may be because private hospitals focused more on cleanliness.

**Table-III:** Level of Precautionary Measures.

A) Precautionary Measures (recipients’ response was agree) Number(n)	P1* 648 (100%)	P2* 648 (100%)	P3* 427 (65.9%)	P4* 617 (95.22%)	P5* 628 (96.91%)	P6* 505 (77.93%)
***Gender***						
Male	548 (84.6%)	548 (84.6%)	353 (54.48%)	523 (80.71%)	533 (82.3%)	431 (66.51%)
Females	100 (15.43%)	100 (15.43%)	74 (11.42%)	94 (14.51%)	95 (14.7%)	74 (11.42%)
Age	31-40 years	31-40	21-30	21-30	51 and above	21-30
***Type of hospital***	187 (28.9%)	187 (28.9%)	140 (21.6%)	181(27.93%)	185 (28.54%)	147 (22.7%)
Public	461 (71.14%)	461 (71.14%)	287 (44.3%)	486 (75%)	443 (68.4%)	358 (55.25%)
Private						

P1* I leave house less frequently than before, P2* I try to maintain a distance of 2-6 feet when in gathering, P3* I did not attend my scheduled visit with doctor due to corona, P4* I wear mask when I leave home, P5* I wash my hands more frequently than before, P6* I leave my shoes outside when I come home.

**Table-IV:** Association of Age, Gender, type of transplant and hospital to fear.

Variables	Significance (p Value)	Test used
Age	X^2^=11.708, p=0.020 < 0.05.	Kruskal wallis test
Gender	P= 0.818 >0.05.	Mann- Whitney U test
Type of transplant	P= 0.747 >0.05.	Mann Whitney U test
Type of hospital Greater for recipients operated in public hospitals than private	U = 36167.50.0, p = .000, effect size= r = 0.13 (small)	Mann Whitney U test

**Table-V:** Association of fear and adherence to precautionary measures.

Adherence to precautionary measure	Significance (p Value)	Test used
1) Those who did not attend in person clinic	U = 37286.0, p = .000, effect size= r = 0.17	Mann Whitney U test
2) Those who wore masks	U = 4537.50.0, p = .000, effect size= r = 0.20.	Mann Whitney U test
3) Those who left their shoes outside their house	U = 3991.50 p = .000, effect size= r = 0.30.	Mann Whitney U test

### Adherence to precautionary measures:

The adherence to precautionary measures was assessed using the second part of the questionnaire. The data was collected as YES (effective) and NO (even). All patients avoided going out unnecessarily and maintained social distance. It was noted that 77.9% number of patients left their shoes outside their home as compared to majority who wore masks and washed their hands more frequently. However, it was also noted that, fear is greater for those patients who didn’t attend in-person clinics (Mdn=4), wear masks (Mdn= 4.5) and left their shoes outside their houses (Mdn= 4.5). Among these, patients who left their shoes outside their houses showed highest effect size.

We used practices like wearing face masks, washing hands, leaving shoes outside the house and preference for on line consultation to see if there was an association, since the maintaining social distance was followed by all. Although, all the practices had small effect size (less than 0.5) but among all the four practices, respondents leaving shoes outside the house (P6*) had large effect size as compared to other practices, depicting that such patients were more conscious during COVID and followed COVID related SOPs more religiously than others.

### Secondary Outcomes:

The effect of COVID- 19 on the health of the post-transplant recipients was assessed through biochemical tests and few direct health related questions. About 78% of patient felt that their health during COVID-19 pandemic improved and while 88% of respondents agreed that their health remained the same during the pandemic. Most recipients (98%) confessed to be compliant to their medication. The biochemical tests showed that 47.2% and 31% of recipients had normal and mild (creatinine level levels below1.5mg/dl) deranged creatinine levels respectively. While almost 96% recipients showed normal liver function tests. Eighty one percent of recipients had HbA1C levels between 6-6.5%.

## DISCUSSION

The clinical and biochemical outcomes of COVID -19 infection in immunocompromised patients was compared to general population^17^ recently. It was noted that they were at high risk of severe infection because of associated co morbid conditions and needed more intensive care and increased mortality.^7^,^18^,^19^ Fernandez-Ruiz et al also reported a case series of 18 SOT with COVID-19 infection with median age of 71 years +-12.8 years and male predominance and fatality rate of 27.8%. Information regarding the effects of COVID -19 infection are scarce and has led to change in the behavior and lives of this special group of patients. Although a lot of work has been done on the attitude and practices of health care workers^20^ we aimed to study the fears, perceptions of this special group of patients during this period.

In Pakistan approximately 975,092 confirmed COVID 19 cases with 222,597 deaths have been reported till date.^21^ It was in late February 2020 that two cases of COVID -19 infection were diagnosed for the first time in Pakistan. Pakistan faces unique challenge of highly porous borders sandwiched between two epicenters of COVID infections and large influx of immigrants from countries with high infection rates (USA, UK), which led to initial rise in COVID cases. Strict nationwide lockdown with border closure and suspension of international flights helped curtail the number. This time period coincided with the first wave of covid-19 infection in Pakistan.^22^,^23^ Punjab one of the most densely populated provinces, with its capital Lahore reported 11,339 cases during the first wave. According to an estimate, there are about 188,655,968 confirmed COVID-19 cases and 4 million deaths world- wide.^21^

During this period the transplantation facilities in Punjab Pakistan were also put on halt as in other transplant centers of the world.^24^,^25^ As reported earlier 26 cases of kidney transplant were on waiting list during first wave of COVID-19 infections.

In our study the median age of recipients who feared most was 31-40 years which is much younger to that mentioned in earlier study^13^, however no relation with the type of transplant was noted. Majority of the recipients were well informed about the disease and feared acquiring the infections from gathering especially the hospitals. This might have been the reason why most of these patients preferred online consultation with their physicians. All patients adhered to precautionary practices like wear masks and frequent hand washing as reported earlier. An even higher level of fear was noted in those patients who left their shoes outside while coming home. SOT recipients take extra care to prevent infections and an increased level of acceptability towards corona SOPs was understandable. The level of fear and anxiety noted amongst this group of patients was more as compared to general Pakistani population during COVID -19 pandemic.^26^

Social media was the main source of information for these patients and only 15% reported special guideline from their treating physicians. This fact may have contributed to heightened fear.^27^,^28^ The patient expressed satisfaction in their present clinical condition despite the pandemic. It was explained in terms of their general health, blood tests, which if not improved remained stable. This may be attributable to enhanced drug compliance seen amongst this special group.

### Limitation of the Study:

This was a cross sectional survey done during the first wave of covid-19 pandemic, the novelty of the virus along with bombardment of information on social media might have resulted in overestimation of fear level. Since data was collected telephonically bias might have been encountered due to lack of interest on part of patients and their attendant. The first wave of COVID -19 infection was relatively mild, however the results might differ if data was collected during second and third wave of COVID-19 pandemic.

## CONCLUSION

An increased level of fear related to COVID-19 pandemic was observed amongst SOT patients during first wave of covid-19 pandemic in Pakistan. This increase level of fear compelled these patients to follow the COVID-19 related infection control measures leading to better health outcomes. However, it also expressed the increased need to develop local expert guidelines for SOT procedures and to ensure improved standard operations against COVID infection at hospitals.

### Authors Contribution:

**AM:** Conceived, designed, data collection and interpretation of data along with writing (drafting) of manuscript.

**HN:** Did conceive, designed, data collection and manuscript editing.

**KI:** Did review and final approval of manuscript.

**ZM:** Did statistical analysis and manuscript editing.
